# Is activated partial thromboplastin time prolongation an independent predictor of bleeding in patients with Kyasanur Forest Disease? A retrospective cohort study from southern India

**DOI:** 10.1016/j.nmni.2026.101808

**Published:** 2026-07-04

**Authors:** Nitin Gupta, Anjely Sebastian, Pothumarthy Venkata Swathikiran, Muralidhar Varma, Chiranjay Mukhopadhyay, Praveen Kumar Tirlangi

**Affiliations:** a Department of Infectious Diseases, Kasturba Medical College, Manipal Academy of Higher Education, Manipal, 576104, India; b Manipal Institute of Virology, Manipal Academy of Higher Education, Manipal, 576104, India; c Department of Microbiology, Kasturba Medical College, Manipal Academy of Higher Education, Manipal, 576104, India

## Abstract

**Background:**

Major bleeding contributes substantially to morbidity in Kyasanur Forest Disease, but predictors of severe hemorrhage remain poorly defined. We aimed to determine whether prolonged baseline activated partial thromboplastin time (APTT) independently predicts major bleeding in patients with Kyasanur Forest Disease.

**Methods:**

We conducted a retrospective cohort study at a tertiary-care referral center in southern India, including adults (≥15 years) hospitalized with RT-PCR–confirmed Kyasanur Forest Disease from January 2019 to May 2025. Major bleeding was defined as a clinically significant bleed involving the gastrointestinal, respiratory, genitourinary, or central nervous systems. Clinical and laboratory parameters were compared between patients with and without major bleeding, with multivariable logistic regression used to assess whether APTT prolongation independently predicted major bleeding.

**Results:**

Among 387 patients with Kyasanur Forest Disease, 39 (10.1%) experienced major bleeding. Gastrointestinal hemorrhage was most common (6.2%), followed by genitourinary bleeding (4.1%). Major bleeders more frequently had thrombocytopenia (97.4% vs 85.9%; p = 0.042), transaminitis (74.4% vs 50.6%; p = 0.005), hypoalbuminemia (54.8% vs 30.8%; p = 0.007), minor mucocutaneous bleeding (17.9% vs 4.6%; p = 0.001), and APTT >40 s (73.3% vs 44.0%; p = 0.002). APTT prolongation remained independently associated with major bleeding (adjusted OR 2.50, 95% CI 1.02–6.14; p = 0.046).

**Conclusion:**

Prolonged APTT at presentation is an independent predictor of significant bleeding in Kyasanur Forest Disease and may serve as a practical marker for early hemorrhagic risk stratification. Prospective validation is warranted to inform clinical decision-making and integrate APTT into severity assessment frameworks.

## Highlights

• Major bleeding occurred in 10% of RT-PCR–confirmed Kyasanur Forest Disease cases.
• Prolonged Activated Partial Thromboplastin Time (APTT) at presentation was strongly associated with major bleeding.
• APTT prolongation independently predicted bleeding after multivariable adjustment.
• Intrinsic coagulation pathway dysfunction contributes to hemorrhagic severity.
• APTT is a simple tool for early bleeding risk stratification in endemic settings.

## 1 Introduction

Kyasanur Forest Disease is a tick-borne viral hemorrhagic fever endemic to the forests of southern India [1]. It was first recognized in 1957 within a confined forested zone of Shivamogga district, Karnataka [2]. Over time, however, the disease has moved well beyond this original hotspot. Human cases and epizootics are now regularly detected across additional districts of Karnataka and in several neighboring states, including Kerala, Tamil Nadu, Goa, and Maharashtra [[3], [4], [5]]. This shift from a local zoonotic illness to a broader regional threat is thought to be linked to deforestation, habitat fragmentation, and increasing human activity along forest margins, all of which bring people into closer contact with infected ticks and animal hosts [[6], [7], [8]]. As Kyasanur Forest Disease continues to emerge in previously unaffected areas, timely recognition of patients at risk of severe disease becomes increasingly important to strengthen clinical care and public health responses.

**Fig. 1:**
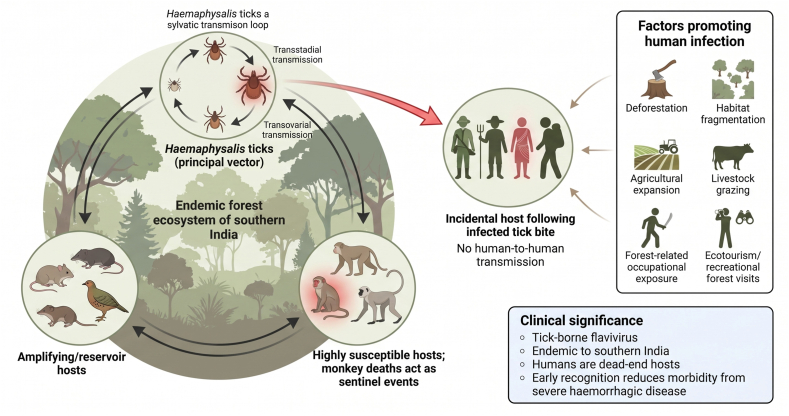
Natural transmission cycle of Kyasanur Forest Disease virus. Natural transmission cycle of Kyasanur Forest Disease virus in the forest ecosystems of southern India. The virus is maintained in a sylvatic transmission cycle involving *Haemaphysalis* ticks and susceptible vertebrate hosts. Small mammals serve as important reservoirs and amplifying hosts, while non-human primates are highly susceptible amplifying hosts whose deaths often act as sentinel events preceding human outbreaks. Humans are incidental dead-end hosts who acquire infection following tick bites during occupational or recreational exposure in forested areas. Environmental changes such as deforestation, habitat fragmentation, agricultural expansion, livestock grazing, and increased human activity at forest margins facilitate spillover into humans. *Created using BioRender.com.*

Kyasanur Forest Disease virus is a member of the genus *Flavivirus* (family *Flaviviridae*) and is maintained in nature through a complex enzootic cycle involving *Haemaphysalis* ticks, small mammals, birds, and non-human primates [1]. Humans are incidental hosts who typically acquire infection following tick bites during occupational or recreational exposure in forested areas (Fig. 1) [1]. Epizootics among monkeys, particularly bonnet macaques and langurs, often precede human outbreaks and serve as important sentinel events for disease surveillance [1]. Although person-to-person transmission has not been documented, the expanding distribution of infected tick populations has increased the risk of infection among forest workers, farmers, tribal communities, military personnel, and travelers visiting endemic regions [1].

The disease is characterized by an acute febrile illness associated with thrombocytopenia, hepatic dysfunction, and varying degrees of hemorrhagic manifestations [1]. Most patients recover with supportive care; however, a subset develops severe disease with significant bleeding, neurological involvement, multiorgan dysfunction, or shock, resulting in substantial morbidity and occasional mortality. While bleeding is a recognized complication of Kyasanur Forest Disease, its frequency and severity are highly variable, and the pathophysiological predictors of major hemorrhage remain poorly understood [9].

Identifying early warning indicators of severe bleeding is crucial because hemorrhagic complications are associated with clinically significant deterioration, including multiorgan involvement and a higher risk of death. Although platelet count abnormalities and hepatic dysfunction have been reported in Kyasanur Forest Disease, limited studies have systematically evaluated coagulation parameters, such as activated partial thromboplastin time (APTT), to assess their prognostic significance [9].

Given that APTT reflects intrinsic coagulation pathway abnormalities and consumptive coagulopathy, key mechanisms implicated in viral hemorrhagic syndromes, its evaluation may help in early risk stratification. This study was undertaken to determine whether prolongation of APTT independently predicts major bleeding in patients with Kyasanur Forest Disease, after adjusting for other potentially relevant clinical and laboratory parameters.

## 2 Methodology

This retrospective cohort study was conducted at a tertiary-care referral center in southern India and included all patients aged 15 years or older who were admitted with RT-PCR–confirmed Kyasanur Forest Disease between January 2019 and May 2025. The study was conducted after obtaining approval from the Institutional Ethics Committee, Kasturba Medical College, Manipal (IEC 1:396/2025).

Patients were categorized into two groups based on whether they developed major bleeding during hospitalization. Major bleeding was defined as clinically significant internal hemorrhage involving organ systems, including upper or lower gastrointestinal bleeding, central nervous system hemorrhage, hemoptysis, or significant genitourinary bleeding such as gross hematuria or severe menstrual bleeding. Minor mucocutaneous bleeding manifestations, including petechiae, purpura, ecchymosis, gum oozing, epistaxis, and streak-like or spot-like bleeds, were not classified as major bleeding.

For the primary analysis, we aimed to evaluate whether APTT prolongation at baseline independently predicted major bleeding. Assuming a major bleeding prevalence of 10%, we planned to include five predictor variables in the final multivariable model [9,10]. Based on a minimum of 7 events per predictor parameter, at least 35 major bleeding events were required. Allowing for up to 10% missing data, the minimum sample size was estimated at 385 patients.

Demographic characteristics, clinical features, laboratory parameters (hematological indices, liver and renal function tests, coagulation profile, inflammatory markers, and muscle injury biomarkers), and outcomes were extracted from medical records. Coagulation abnormalities were defined as an APTT greater than 40 s and an international normalized ratio (INR) of 1.5 or higher. Transaminitis was defined as serum transaminase levels greater than 200 IU/L.

Categorical variables were compared using Chi-square or Fisher's exact tests and continuous variables using independent samples *t*-test or Mann–Whitney *U* test as appropriate. Statistical significance was set at p < 0.05. Univariable analyses were followed by multivariable binary logistic regression with major bleeding as the dependent variable. Significant variables from univariable analysis were considered for model inclusion. To prevent overfitting, a step-wise exclusion strategy was used to retain no more than five predictors, prioritizing those with clinical relevance and robust univariable associations. Model calibration and performance were assessed using the Omnibus test, Nagelkerke R^2^ statistic, and the Hosmer–Lemeshow goodness-of-fit test. Analyses were performed using IBM SPSS Statistics version 26.

## 3 Results

A total of 387 patients met the inclusion criteria. Major bleeding occurred in 39 patients (10.1%). Among the 387 confirmed Kyasanur Forest Disease cases in this cohort, gastrointestinal bleeding was the most common hemorrhagic manifestation, occurring in 24 patients (6.2%). Genitourinary bleeding was documented in 16 patients (4.1%), while respiratory tract hemorrhage in the form of hemoptysis was noted in 3 patients (0.8%). Central nervous system bleeding was rare, observed in only two patients (0.5%).

Age and sex were comparable between groups. Common presenting clinical features, such as myalgia, headache, rash, conjunctival suffusion, abdominal pain, loose stools, cough, lower respiratory tract involvement, altered sensorium, and seizures, did not differ significantly between those who developed major bleeding and those who did not (Table 1). Minor mucocutaneous bleeding was more frequently observed in patients who progressed to major bleeding (17.9% vs 4.6%, p = 0.001). Thrombocytopenia was significantly more common in major bleeders (97.4% vs 85.9%, p = 0.042). Markers of hepatic involvement were strongly associated with major bleeding: transaminitis (>200 IU/L) and hypoalbuminemia (<3.5 g/dL) were significantly more frequent among major bleeders (p = 0.005 and p = 0.007, respectively). APTT >40 s was present in 73.3% of major bleeders compared with 44.0% of non-bleeders (p = 0.002), and INR ≥1.5 in 6.3% versus 0.3% (p = 0.001). Elevated creatinine, creatine phosphokinase, and C-reactive protein were not associated with major bleeding.

**Table 1:** Comparison of factors associated with bleeding in patients with Kyasanur Forest Disease.

Variable	Major Bleeds (n = 39)	No Major Bleed (n = 348)	p-value
Sex (Male)	23/39 (59.0%)	214/348 (61.5%)	0.759
Myalgia	21/39 (53.8%)	155/348 (44.5%)	0.268
Headache	11/39 (28.2%)	121/348 (34.8%)	0.412
Rash	5/39 (12.8%)	23/348 (6.6%)	0.156
Conjunctival suffusion	8/39 (20.5%)	44/348 (12.6%)	0.172
Lymphadenopathy	4/39 (10.3%)	13/348 (3.7%)	0.060
Abdominal pain	3/39 (7.7%)	58/348 (16.7%)	0.145
Loose stools	12/39 (30.8%)	68/348 (19.5%)	0.101
Cough	2/39 (5.1%)	39/348 (11.2%)	0.242
LRT involvement	8/39 (20.5%)	43/348 (12.4%)	0.153
Altered sensorium	6/39 (15.4%)	56/348 (16.1%)	0.909
Seizure	2/39 (5.1%)	20/348 (5.7%)	0.874
Minor mucocutaneous bleeding (MCB)	7/39 (17.9%)	16/348 (4.6%)	**0.001**
Leucopenia	5/39 (12.8%)	55/346 (15.9%)	0.616
Thrombocytopenia	38/39 (97.4%)	298/347 (85.9%)	**0.042**
Transaminitis (>200)	29/39 (74.4%)	176/348 (50.6%)	**0.005**
Hypoalbuminemia <3.5 g/dL	17/31 (54.8%)	86/279 (30.8%)	**0.007**
APTT >40 s	22/30 (73.3%)	121/275 (44.0%)	**0.002**
INR ≥1.5	2/32 (6.3%)	1/287 (0.3%)	**0.001**
Elevated CRP	5/22 (22.7%)	34/206 (16.5%)	0.461
Elevated Creatinine >1.2 mg/dL	8/38 (21.1%)	62/347 (17.9%)	0.629
Elevated CPK	22/26 (84.6%)	199/216 (92.1%)	0.198

Major bleeding refers to clinically significant internal hemorrhage involving gastrointestinal, respiratory, genitourinary, or central nervous system sites. Minor mucocutaneous bleeding (MCB) includes petechiae, purpura, gum bleeding, and epistaxis. Thrombocytopenia was defined as a platelet count <150 × 10^3^/μL. Transaminitis was defined as serum aminotransferase levels greater than 200 IU/L. Hypoalbuminemia was defined as serum albumin <3.5 g/dL. APTT indicates activated partial thromboplastin time, with APTT >40 s indicating an intrinsic coagulation pathway abnormality. INR indicates international normalized ratio, with INR ≥1.5 indicating prolonged prothrombin time. CPK indicates creatine phosphokinase, and CRP indicates C-reactive protein. p-values calculated using Chi-square test or Fisher's exact test as appropriate.

On multivariable logistic regression analysis, APTT >40 s remained an independent predictor of major bleeding after adjustment for other significant univariable associations (adjusted OR: 2.50, 95% CI: 1.02–6.14, p = 0.046) (Table 2). Minor mucocutaneous bleeding also independently predicted major bleeding (adjusted OR: 3.42, 95% CI: 1.02–11.46, p = 0.046). Thrombocytopenia, hypoalbuminemia, and elevated INR did not retain significance in the adjusted model. The final multivariable model demonstrated good overall performance (Omnibus χ^2^ = 22.13, p < 0.001; Nagelkerke R^2^ = 0.150; Hosmer–Lemeshow p = 0.970).

**Table 2:** Multivariable logistic regression analysis identifying independent predictors of major bleeding in patients with Kyasanur Forest Disease.

Variable	OR	aOR	95% CI	p-value
APTT >40 s	**3.5 (1.5**–**8.1)**	**2.50**	**1.02**–**6.14**	**0.046**
Elevated INR ≥1.5	19.1 (1.7-216.5)	9.54	0.35 – 258.38	0.180
Hypoalbuminemia <3.5 g/dL	2.7 (1.3-5.8)	1.80	0.79 – 4.13	0.162
Minor mucocutaneous bleeding	4.5 (1.7-11.8)	**3.42**	**1.02**–**11.46**	**0.046**
Thrombocytopenia	6.2 (0.8-46.6)	3.23	0.35 – 29.98	0.302

Adjusted odds ratios (aOR) and 95% confidence intervals (CI) are shown for variables included in the final model. Major bleeding served as the dependent outcome. APTT indicates activated partial thromboplastin time; INR, international normalized ratio; MCB, minor mucocutaneous bleeding. Variables were selected based on significant associations in univariable analysis and included through a step-wise model-reduction approach to maintain events-per-predictor adequacy.

## 4 Discussion

In this retrospective cohort of 387 RT-PCR–confirmed Kyasanur Forest Disease patients, major bleeding occurred in 39 individuals (10.1%). Gastrointestinal hemorrhage was the most frequent site, followed by genitourinary and respiratory involvement. Patients with major bleeding showed significantly higher rates of thrombocytopenia, transaminitis, hypoalbuminemia, minor mucocutaneous bleeding, and APTT prolongation compared with those without bleeding. Among these variables, an APTT greater than 40 s remained independently associated with major hemorrhage in multivariable logistic regression analysis, indicating that intrinsic pathway coagulation abnormalities were strongly linked to bleeding severity in this cohort.

Hemorrhagic manifestations are a significant clinical component of Kyasanur Forest Disease, although their frequency varies across outbreaks [4,9,11]. In a previous outbreak in Maharashtra, bleeding was observed in 28.5% of cases [4]. In another study from Kerala, bleeding was observed in only 8% of cases [12]. Consistent with our observations, earlier studies have shown that gastrointestinal bleeding is among the most prominent internal hemorrhagic features, typically presenting as hematemesis or melena, and often serving as the earliest clinical sign of progression to severe disease [4,13,14]. Respiratory tract bleeding, manifesting as hemoptysis, has been described particularly in patients with advanced illness, while genitourinary bleeding, including hematuria and, less frequently, per-vaginal bleeding, has also been reported [4,9,11,13]. Minor mucocutaneous bleeding, such as epistaxis, gum bleeding, and petechiae, has been widely documented and may precede internal hemorrhage, acting as a clinical indicator of evolving coagulopathy. Taken together, the available literature supports that bleeding in Kyasanur Forest Disease spans a spectrum from mild mucosal involvement to life-threatening internal hemorrhage, underscoring the importance of early recognition of patients at heightened risk.

The mechanisms contributing to hemorrhage in Kyasanur Forest Disease appear multifactorial and interlinked. First, direct endothelial injury plays a crucial role: Kyasanur Forest Disease Virus infects and activates vascular endothelial cells, stimulating inflammatory pathways and disrupting intercellular junctions, thereby increasing vascular permeability and predisposing to plasma leakage and bleeding [15,16]. Second, thrombocytopenia is commonly observed early in the disease course and likely contributes by reducing primary hemostasis, thereby increasing susceptibility to mucosal and internal bleeding [9,13]. Third, hepatic involvement may increase the risk of bleeding [17]. Transaminitis and hypoalbuminemia were significantly overrepresented among patients with major bleeding in our cohort, consistent with the hepatotropic nature of Kyasanur Forest Disease Virus [12]. Autopsy studies describe parenchymal degeneration in major organs, including the liver [18]. Hepatocellular injury may impair the synthesis of coagulation factors, alter fibrinogen production, and promote vascular fragility through the activity of inflammatory cytokines [17]. However, liver dysfunction cannot fully explain the coagulopathy observed, as prothrombin time/international normalized ratio (PT/INR) abnormalities were uncommon in this study. A fourth and increasingly recognized contributor is selective intrinsic pathway dysfunction, reflected by the predominance of APTT prolongation with relatively preserved PT/INR [9]. In our cohort, both APTT and PT/INR were associated with bleeding on univariate analysis, but prolonged INR was uncommon and did not remain significant in multivariable analysis. The disproportionately prolonged APTT suggests targeted intrinsic pathway derangement rather than global coagulopathy. Evidence from our earlier Karnataka cohort supports this concept, in which APTT prolongation was observed in over half of patients during the acute phase and normalized during recovery, indicating a transient, virus-induced coagulopathy rather than chronic hepatic impairment [9,19]. Finally, intrinsic factor depletion, particularly Factor IX suppression, may be a key mechanistic driver. A recent case from our center demonstrated reversible Factor IX deficiency during acute infection, confirmed through mixing studies that showed correction in normal and aged plasma but not in adsorbed plasma [20]. The subsequent recovery of both APTT and Factor IX levels further strengthens the hypothesis of virus-mediated intrinsic factor consumption or inhibition rather than congenital deficiency.

Our findings have important clinical implications, particularly in endemic, resource-limited settings where access to advanced diagnostic tools may be limited. Activated partial thromboplastin time is inexpensive, rapidly measurable, and widely available as part of routine coagulation testing. Incorporating the APTT into the routine evaluation of patients with Kyasanur Forest Disease may facilitate early identification of those at increased risk of major bleeding, allowing closer monitoring, timely preparation for blood product support, and referral to higher-level care when indicated. These findings are also relevant beyond endemic regions. As Kyasanur Forest Disease continues to expand geographically within southern India and travel to endemic forested areas increases, clinicians practicing elsewhere may increasingly encounter returning travelers with acute febrile illness following forest exposure. In such settings, where familiarity with the disease may be limited, prolonged activated partial thromboplastin time is a simple, readily available marker that supports early hemorrhagic risk stratification and guides appropriate supportive management. Given the absence of specific antiviral therapy, early identification of patients at increased risk of severe hemorrhagic complications remains essential to optimize clinical outcomes.

Strengths of this study include a relatively large cohort with confirmed infection and clear clinical outcome definitions. Limitations include its retrospective design, potential for documentation bias in bleeding classification, and absence of detailed virological severity markers. Prospective studies should examine longitudinal APTT kinetics to evaluate temporal prediction accuracy and validate its incorporation into clinical severity scoring tools.

## 5 Conclusion

Prolongation of APTT is an independent predictor of major bleeding in patients with Kyasanur Forest Disease, highlighting its value as an early and readily accessible marker of severe disease. Minor mucocutaneous bleeding may serve as an early clinical indicator of progression toward major hemorrhage. Incorporating APTT assessment into routine evaluation and monitoring of Kyasanur Forest Disease could improve risk stratification and guide timely intervention. Further prospective validation is warranted to strengthen the application of this approach in clinical practice.

## Data sharing

De-identified patient data from this study are held at the Department of Infectious Diseases, Kasturba Medical College, Manipal. The dataset may be made available by the corresponding author on reasonable request, subject to institutional approvals and data-sharing regulations.

## Ethics approval

This study was approved by the Institutional Ethics Committee of Kasturba Medical College, Manipal (IEC 1:396/2025). As a retrospective analysis of anonymised clinical data, informed consent requirements were waived.

## Funding

This research received no external funding.

## CRediT authorship contribution statement

**Nitin Gupta:** Conceptualization, Data curation, Formal analysis, Investigation, Methodology, Software, Supervision, Validation, Writing – original draft, Writing – review & editing. **Anjely Sebastian:** Data curation, Investigation, Methodology, Project administration, Writing – original draft, Writing – review & editing. **Pothumarthy Venkata Swathikiran:** Data curation, Investigation, Writing – original draft, Writing – review & editing. **Muralidhar Varma:** Project administration, Supervision, Writing – review & editing. **Chiranjay Mukhopadhyay:** Investigation, Methodology, Project administration, Writing – review & editing. **Praveen Kumar Tirlangi:** Conceptualization, Validation, Visualization, Writing – original draft, Writing – review & editing.

## Declaration of competing interest

The authors declare that they have no known competing financial interests or personal relationships that could have appeared to influence the work reported in this paper.

## References

[1] Gupta N., Wilson W., Neumayr A., Saravu K. (2022). Kyasanur forest disease: a state-of-the-art review. QJM.

[2] Work T.H. (1958). Virological aspects of Kyasanur Forest disease. J Indian Med Assoc.

[3] Awate P., Yadav P., Patil D. (2016). Outbreak of Kyasanur Forest disease (monkey fever) in Sindhudurg, Maharashtra State, India, 2016. J Infect.

[4] Gurav Y.K., Yadav P.D., Gokhale M.D. (2018). Kyasanur Forest disease prevalence in Western ghats proven and confirmed by recent outbreak in Maharashtra, India. Vector Borne Zoonotic Dis.

[5] Oliveira A., Selvaraj K., Tripathy J.P. (2020). Geospatial clustering, seasonal trend and forecasting of Kyasanur Forest Disease in the state of Goa, India, 2015-2018. Trop Med Health.

[6] Walsh M.G., Bhat R., Nagarajan-Radha V. (2021). Low mammalian species richness is associated with Kyasanur Forest disease outbreak risk in deforested landscapes in the Western Ghats, India. One Health.

[7] Walsh M.G., Mor S.M., Maity H., Hossain S. (2020). A preliminary ecological profile of Kyasanur Forest disease virus hosts among the mammalian wildlife of the Western Ghats, India. Ticks Tick Borne Dis.

[8] Walsh M.G., Mor S.M., Maity H., Hossain S. (2019). Forest loss shapes the landscape suitability of Kyasanur Forest disease in the biodiversity hotspots of the Western Ghats, India. Int J Epidemiol.

[9] Gupta N., Chunduru K., Safeer K.M., Saravu K. (2021). Clinical and laboratory profile of patients with Kyasanur forest disease: a single-centre study of 192 patients from Karnataka, India. J Clin Virol.

[10] Riley R.D., Ensor J., Snell K.I.E., Harrell F.E., Martin G.P., Reitsma J.B., Moons K.G.M., Collins G., van Smeden M. (2020). Calculating the sample size required for developing a clinical prediction model. Br Med J.

[11] Webb H.E., Rao R.L. (1961). Kyasanur forest disease: a general clinical study in which some cases with neurological complications were observed. Trans R Soc Trop Med Hyg.

[12] Sadanandane C., Elango A., Marja N., Sasidharan P.V., Raju K.H.K., Jambulingam P. (2017). An outbreak of Kyasanur forest disease in the Wayanad and Malappuram districts of Kerala, India. Ticks Tick Borne Dis.

[13] Pavri K. (1989). Clinical, clinicopathologic, and hematologic features of Kyasanur Forest disease. Rev Infect Dis.

[14] Tandale B.V., Balakrishnan A., Yadav P.D., Marja N., Mourya D.T. (2015). New focus of Kyasanur Forest disease virus activity in a tribal area in Kerala, India, 2014. Infect Dis Poverty.

[15] Holbrook M.R. (2012). Kyasanur forest disease. Antivir Res.

[16] Sirmarova J., Salat J., Palus M. (2018). Kyasanur Forest disease virus infection activates human vascular endothelial cells and monocyte-derived dendritic cells. Emerg Microb Infect.

[17] Flores B., Trivedi H.D., Robson S.C., Bonder A. (2017). Hemostasis, bleeding and thrombosis in liver disease. J Transl Sci.

[18] Iyer C.G., Laxmana Rao R., Work T.H., Narasimha Murthy D.P. (1959). Kyasanur Forest Disease VI. Pathological findings in three fatal human cases of Kyasanur Forest Disease. Indian J Med Sci.

[19] Gupta N., Varma M., Saravu K. (2020). Difference in clinical presentation between the first and second phases of Kyasanur Forest disease: an experience from a teaching hospital in South India. Inf Med.

[20] Kumar T.P., Belurkar S., Varma M., Kiran P.V.S., Gupta N. (2025). Mixing studies in viral haemorrhagic fever. QJM.

